# A case report of a rare cardiac anomaly associated with Ellis–van Creveld syndrome: common atrium, partial atrioventricular septal defect, and hypoplastic left ventricle

**DOI:** 10.1093/ehjcr/ytaf005

**Published:** 2025-01-10

**Authors:** Takashi Kido, Shuhei Toba, Takato Yamasaki, Dai Asada, Sanae Tsumura

**Affiliations:** Department of Cardiovascular Surgery, Osaka University Graduate School of Medicine, Suita, Osaka 565-0871, Japan; Department of Thoracic and Cardiovascular Surgery, Mie University Graduate School of Medicine, Tsu, Mie 514-8507, Japan; Department of Thoracic and Cardiovascular Surgery, Mie University Graduate School of Medicine, Tsu, Mie 514-8507, Japan; Department of Cardiovascular Surgery, Osaka Women’s and Children’s Hospital, Izumi, Osaka 594-1101, Japan; Department of Pediatric Cardiology, Osaka Women’s and Children’s Hospital, Izumi, Osaka 594-1101, Japan

**Keywords:** Ellis–van Creveld syndrome, Common atrium, Partial atrioventricular septal defect, Perfusion–distention fixation, Case report

## Abstract

**Background:**

A partial atrioventricular septal defect (AVSD) with a hypoplastic left ventricle and common atrium is a rare combination of cardiac anomalies that can be associated with Ellis–van Creveld (EVC) syndrome.

**Case summary:**

A female neonate with EVC syndrome was diagnosed with an unbalanced AVSD and hypoplastic left ventricle. Pulmonary artery banding and ductus ligation were performed at 23 days after birth. The postoperative course was complicated by moderate atrioventricular valve (AVV) regurgitation and low cardiac output. On postoperative Day 6, emergent extracorporeal membrane oxygenation (ECMO) was performed to treat acute circulatory failure. Under suspicion of systemic ventricular outflow tract obstruction, the Damus–Kaye–Stansel procedure with a systemic-to-pulmonary artery shunt and AVV plasty were performed. Intraoperatively, no ventricular septal defect was found. The right and left AVV orifices were found to be separated. Postoperatively, the patient could not be weaned from cardiopulmonary bypass and continued to receive ECMO support. Eight days postoperatively, a right ventricle-to-pulmonary artery shunt and division of the systemic-to-pulmonary artery shunt were performed to increase the pulmonary blood flow. On postoperative Day 5, the ECMO was successfully removed under continuous infusion of adrenalin, but the patient died of severe renal failure 4 days later. The parents consented to autopsy. The heart was permanently preserved by perfusion–distention fixation and wax infiltration.

**Discussion:**

We reported a rare combination of cardiac defects of common atrium, partial AVSD, and hypoplastic left ventricle associated with EVC syndrome. Accurately diagnosing the presence of ventricular septal defect is essential part in determining surgical treatment strategy.

**Learning points**

- The rare combination of cardiac defects, common atrium, partial atrioventricular septal defect, and hypoplastic left ventricle, can be associated with Ellis–van Creveld syndrome.
- Perfusion–distention fixation and wax infiltration are effective to generate a stable and solid specimen allowing detailed examination of the anatomical features of cardiac malformation.

## Introduction

Ellis–van Creveld (EVC) syndrome is an autosomal recessive disorder characterized by clinical manifestations of short-limb dwarfism, postaxial polydactyly, abnormalities in tooth and nail development, thoracic dystrophy, and congenital heart disease. Cardiac malformations reportedly occur in ∼60%–74% of patients with EVC syndrome.^1^ While the spectrum of cardiac abnormalities is broad, a common atrium with atrioventricular septal defect (AVSD) occurs frequently in patients with EVC syndrome.

We herein describe a patient with a partial AVSD, hypoplastic LV, and common atrium associated with EVC syndrome who died after surgical treatment. The patient’s heart was preserved by perfusion–distention fixation and wax infiltration to generate a stable and solid specimen allowing detailed examination of the anatomical features of this rare cardiac malformation.

## Summary figure

**Figure:**
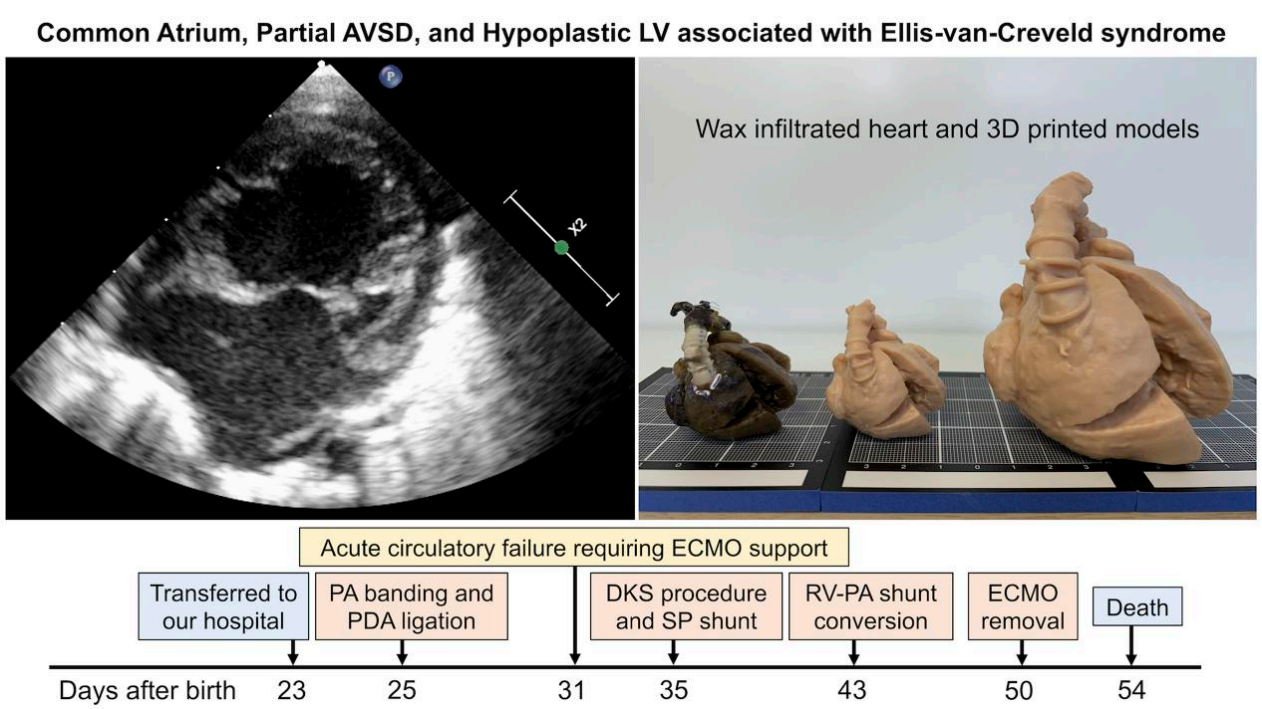


## Case presentation

**Figure 1:**
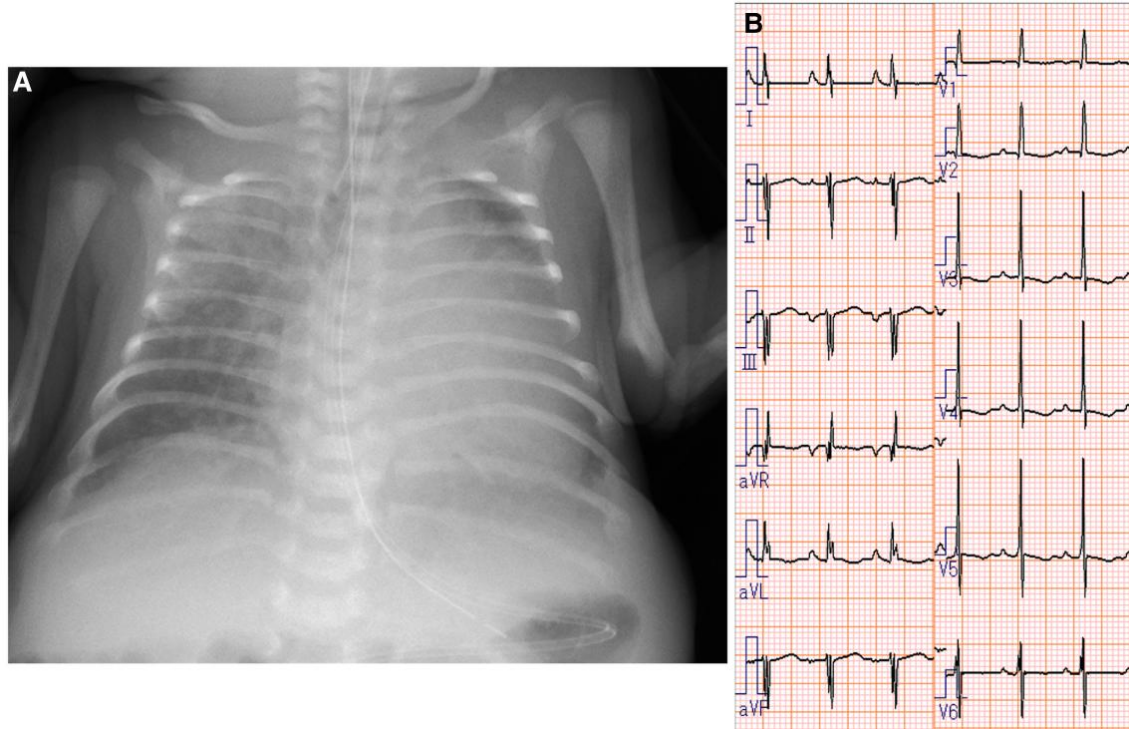
Chest X-ray (*A*) and electrocardiogram findings (*B*) on admission.

**Figure 2:**
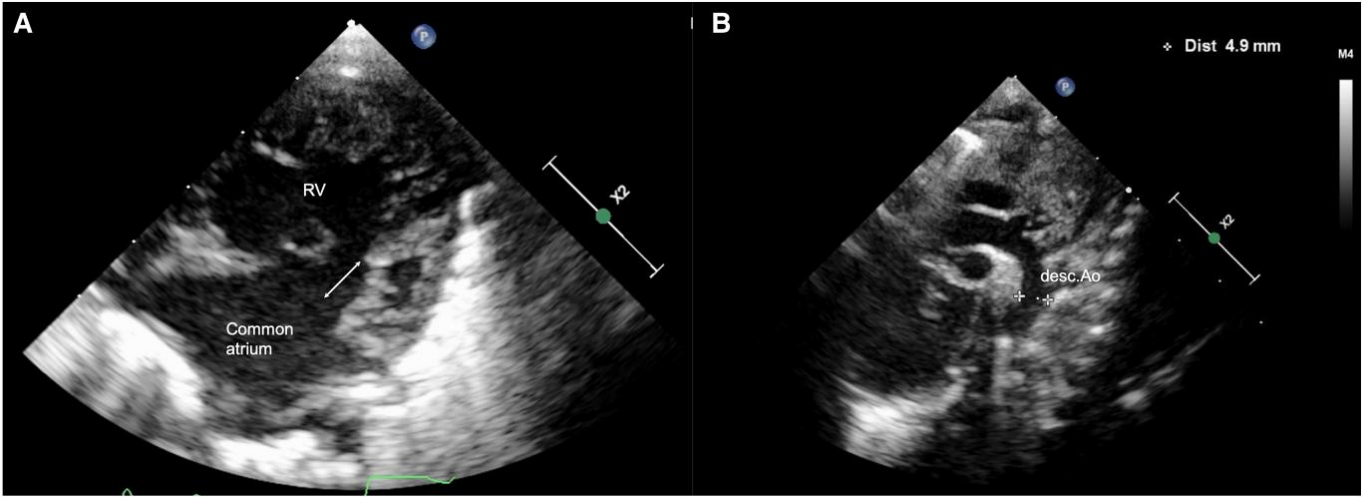
Echocardiogram findings on admission showing four-chamber view (*A*), aortic arch (*B*). White arrow indicates suspected ventricular septal defect. RV, right ventricle; desc.Ao, descending aorta.

A female neonate was born at 33 weeks’ gestation weighing 2.8 kg. She was diagnosed with an unbalanced AVSD and hypoplastic left ventricle (LV). She was intubated immediately after birth because of respiratory distress associated with severe generalized oedema and pleural effusion. She had several extracardiac anomalies, including prune belly syndrome, anovestibular fistula, polydactyly, and short-limb dwarfism, the last two of which are typical of EVC syndrome. She was transferred to our hospital for further treatment at 23 days after birth. On arrival, the patient’s body weight was 1.9 kg. The chest X-ray showed mild cardiomegaly with pulmonary congestion and the electrocardiogram showed sinus rhythm with left axis deviation (*Figure 1*). We performed echocardiography and diagnosed a common atrium, unbalanced complete AVSD with hypoplastic LV, patent ductus arteriosus, and moderate atrioventricular valve (AVV) regurgitation (*Figure 2*, Supplemental file 1). The diameter of the ventricular septal defect (VSD) was measured as 11 mm (upon later inspection, however, no VSD was present) (*Figure 2*); and bidirectional blood flow was shown in the ductus arteriosus without coarctation of the aorta (Supplemental file 2).

Two days after admission, pulmonary artery (PA) banding and ductus ligation were performed. The postoperative course was complicated by persistent moderate AVV regurgitation, low cardiac output, and the need for continuous adrenalin infusion. On postoperative Day 6, emergent extracorporeal membrane oxygenation (ECMO) was performed to treat acute circulatory failure. Under suspicion of systemic ventricular outflow tract obstruction, the Damus–Kaye–Stansel procedure with a systemic-to-PA shunt and AVV plasty were performed. Intraoperatively, no VSD was found. The right and left AVV orifices were found to be separated. Thus, the correct diagnosis was a partial AVSD. Eight days postoperatively, she could not be weaned from ECMO because of hypoxia. We decided to perform a right ventricle-to-PA shunt and division of the systemic-to-PA shunt, aiming to increase the pulmonary blood flow. Postoperatively, the oxygen saturation level improved and the cardiopulmonary bypass was discontinued under continuous infusion of adrenaline. We decided to continue ECMO support until the cardiac function recovered. On postoperative Day 5, the patient developed acute renal failure. Because of the high probability of renal complications from haemolysis, we decided to remove the ECMO. Although the ECMO was successfully removed under continuous infusion of adrenaline, the renal function did not recover and the patient died 4 days after ECMO removal. The clinical course is summarized in timeline.

**Figure 3:**
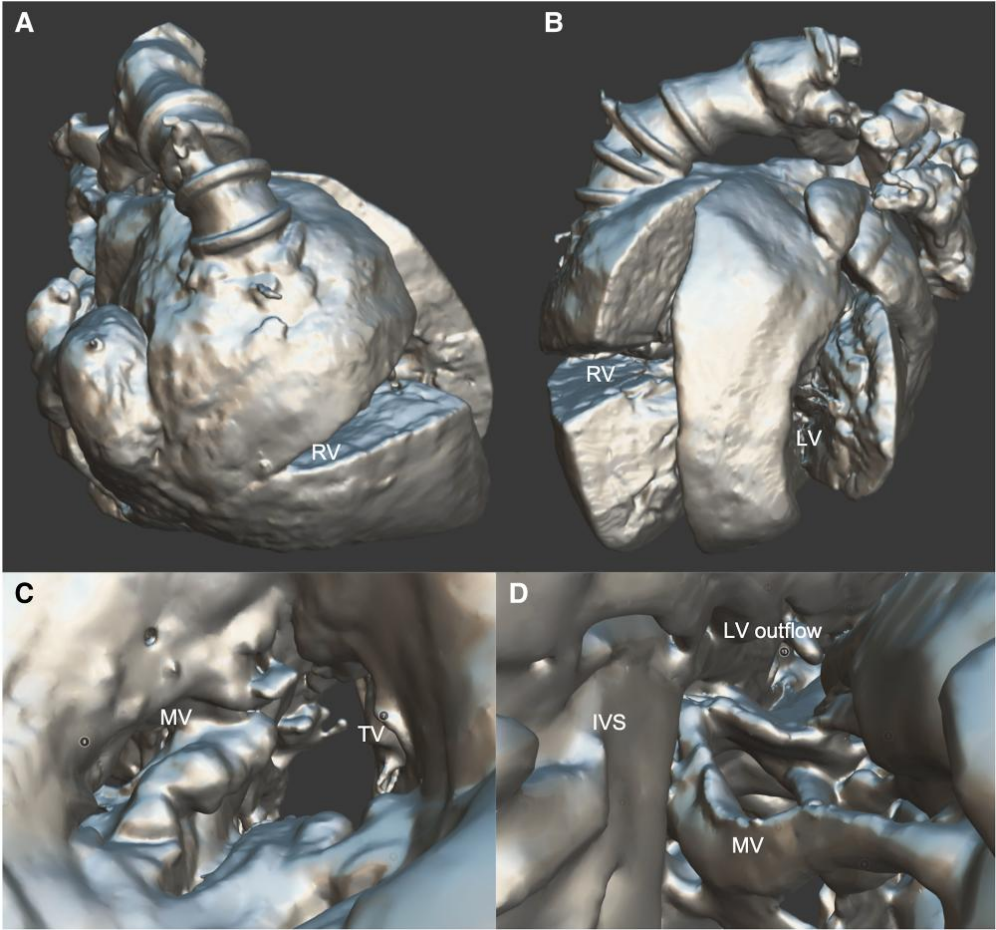
The anterior view (*A*) and lateral view (*B*) of digital 3D model. (*C*) Digital 3D image of the atrioventricular valve as viewed from the common atrium. (*D*) Digital 3D image of the mitral valve, interventricular septum, and left ventricular outflow tract as viewed from the apex of the left ventricle. LV, left ventricle; RV, right ventricle; MV, mitral valve; TV, tricuspid valve; IVS, intraventricular septum.

The parents consented to a heart autopsy. The heart was extracted and preserved using perfusion–distension fixation. The detailed procedure of perfusion–distension fixation is described in a previous report.^2^ After perfusion–distension fixation, we treated the specimen with wax infiltration as previously described. During the wax infiltration, portions of the atrium and ventricular wall were excised to allow observation of the intracardiac anatomy. Micro-focused computed tomography of the wax-infiltrated heart specimen was performed to obtain three-dimensional (3D) data using a CosmoScan GX II system (Rigaku Corp., Tokyo, Japan) with a tube current of 88 µA, tube voltage of 90 kV, and voxel size of 87 µm. Based on the 3D data, a 3D model was digitally reconstructed using Amira (Thermo Fisher Scientific, Waltham, MA, USA), and physical 3D models were created using a 3D printer (Form 3B; Formlabs Inc., Somerville, MA, USA) in both the original size and twice the size of the original. The wax-infiltrated heart specimen and 3D printed models are shown in the graphical abstract. The digital 3D model is available in Supplemental file 3. The appearance of AVV and left ventricle are shown in *Figure 3*.

## Discussion

A partial AVSD with hypoplastic LV and common atrium is a rare combination of cardiac anomalies. Our patient was misdiagnosed with a complete AVSD with hypoplastic LV, and PA banding was therefore performed to control the pulmonary blood flow. As a result, circulatory failure developed secondary to systemic ventricular outflow obstruction. The palliative surgical procedures that could serve as alternatives to PA banding in this patient were the primary DKS procedure or bilateral PA banding. The primary DKS procedure in the neonatal period is technically difficult but can relieve systemic outflow tract obstruction without the deleterious effects of PA banding in patients with single-ventricle physiology. Previous reports have described the advantages of bilateral PA banding and ductal stenting or continuous infusion of prostaglandin E2 in patients with hypoplastic left heart syndrome. This technique could have been applied in our case to maintain systemic output through the right ventricle while the pulmonary over-circulation was relieved. However, the appearance of the cardiac anatomy, normally sized ascending aorta, assumed presence of VSD, and bidirectional flow in the ductus arteriosus prevented us from performing this procedure.

Ellis–van Creveld syndrome is a rare autosomal recessive genetic abnormality that has been linked to a mutation in the *EVC* or *EVC2* gene located on the distal short arm of chromosome 4.^3^ A partial AVSD with a common atrium is often found in patients with EVC syndrome. O’Connor *et al*.^4^ reported nine patients with EVC syndrome who underwent cardiac surgery in the infantile period. Of these nine patients, seven had a balanced AVSD with common atrium (partial AVSD in four, intermediate AVSD in two, and complete AVSD in one).^4^ Hills *et al*.^5^ summarized 92 previously reported cases of EVC syndrome and an additional 32 cases that were identified from a multicentre registry. Among all 124 patients with EVC syndrome, 76 (61%) had an AVSD and 57 (46%) had a common atrium. A hypoplastic LV was present in only five (4%) patients.^5^ In the presence of a hypoplastic LV, a partial AVSD is a very rare cardiac abnormality, and careful diagnosis is recommended in patients with EVC syndrome.

Although we could not save this patient, we successfully preserved the heart specimen by perfusion–distention fixation and produced a wax-infiltrated heart. In contrast to conventional formalin fixation, perfusion–distension fixation is a more ideal method to preserve the 3D cardiac anatomy. The resulting stable and solid heart specimen provides a comprehensive understanding of complex heart anatomy. Moreover, wax-infiltrated specimens can be subjected to computed tomography imaging, yielding 3D data sets for teaching and detailed investigation of the cardiac morphology. Images reconstructed from these 3D data sets can be viewed and manipulated on a screen or be printed with a high-resolution 3D printer to create copies, allowing us to share the understanding of rare cardiac anatomies.

## Lead author biography


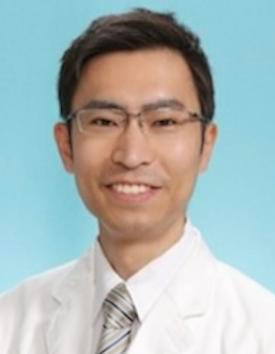


Takashi Kido is a congenital heart surgeon who worked at Boston Children’s Hospital (2019–20) and the German Heart Center Munich (2020–22). He is currently an assistant professor in the Department of Cardiovascular Surgery at the Osaka University Graduate School of Medicine. His specialities include congenital heart surgery, heart transplantation, and adult congenital heart disease.

## Supplementary Material

ytaf005_Supplementary_Data

## Data Availability

The data that support the findings of this study are available from the authors upon reasonable request.

## Notes

**Consent:** The authors confirm that written consent for the submission and publication of this case report, including the images and associated text, has been obtained from the parents of the patient, in line with COPE guidance.

**Funding:** Creation of digital and physical 3D models of the specimen was supported by the Japan Society for the Promotion of Science KAKENHI (21K08860) and the Uehara Memorial Foundation.
